# Local Small Ruminant Grazing in the Monti Foy Area (Italy): The Relationship Between Grassland Biodiversity Maintenance and Added-Value Dairy Products

**DOI:** 10.3389/fvets.2020.546513

**Published:** 2020-11-25

**Authors:** Salvatore Claps, Marisabel Mecca, Adriana Di Trana, Lucia Sepe

**Affiliations:** ^1^Research Centre for Animal Production and Aquaculture, Council for Agricultural Research and Economics, Bella, Italy; ^2^School of Agricultural, Forestry, Food and Environmental Sciences, University of Basilicata, Potenza, Italy

**Keywords:** grazing, mountain, local breed, small ruminant, dairy product quality, biodiversity maintenance

## Abstract

The literature indicates that grazing small ruminants, when adequately managed, contributes to grassland biodiversity maintenance. On the other hand, milk and cheese from grazing animals show higher nutritional and aromatic quality than those from stall-fed animals. The relationship between the two issues has rarely been addressed. This article provides information for a discussion of this relationship. First, two case studies are reported. Local breeds of small ruminants fed by grazing on pastures within the Special Area of Conservation “Monti Foy” in the Northwestern Basilicata region (Italy), with a stocking rate of 4.0 LU ha^−1^ year^−1^, showed the best effectiveness for the maintenance of grassland botanical biodiversity. Milk and cheese from pasture-fed goats showed higher contents of beneficial fatty acids, phenols, and vitamins A and E; higher degree of antioxidant protection; and richer volatile compound profiles, in particular for terpenes content. Finally, some recommendations for the management of grazing systems in similar mountain areas are offered, including a viable approach for land managers to preserve the grassland biodiversity of pastures and provide high-quality products that are valuable both for their nutritional quality and for their contribution to the economic sustainability of mountain communities.

## Introduction

The grazing system has been an important component of the Mediterranean environment for millennia; thus, it represents a valid tool for managing and preserving that environment (1–3). In the Mediterranean environment, various ecosystems coexist, herbaceous, bushy, and woody, and are not always in balance; however, they are prone to rapid recovery and are thus considered very resilient (4, 5).

Rangeland management is generally difficult due to the complexity of the ecosystems, with great diversity in plant communities, soils, and grazing practices (6, 7). Several authors have pointed out the importance of a correct livestock management on overgrazed or undergrazed areas, in order to preserve or increase the floristic richness and the nutritional value of grassland (8–10) and forage and to improve the animal productive performances (milk yield) (11).

Good management of extensive silvopastoral systems could play an important role in the delivery of many ecosystem services, as was recently exhaustively stated by the UK National Ecosystem Assessment (12). In mountain areas characterized by forests/shrubs and meadows, well-managed pastoral activity could be considered a tool for landscape preservation, fire prevention (13), and grassland biodiversity maintenance, contributing to the overall economic benefit of mountain communities.

Grazing behavior is another key factor in specific landscape and pasture biodiversity determinism (14). Grazing behavior has important consequences; in addition to contributing to animal nutrition, it affects the specific characteristics, features, and quality of animal products (milk and dairy products) (15–17). When local breeds are reared in an adequately managed and rational grazing system, they are successful in preserving grassland biodiversity. When they browse the apices and flowers of plants that may be unpalatable for cosmopolitan breeds, local breeds limit the diffusion of various unpalatable and weed species and maintain the floristic balance, thus enhancing the nutritional value of pastures (18).

To protect pastoral areas, the European Union has developed a series of measures (EC Reg. No. 796/04 and subsequent amendments). In particular, Standard 4.6 (“Minimum Livestock Stocking Rate and/or Appropriate Regimes”) aims to “ensure a minimum level of maintenance and avoid the deterioration of habitats” and to protect pastures, especially through avoiding grassland degradation in certain ecologically significant areas [Annex IV of Council Regulation (EC) No. 1782/2003].

In the central area of the Basilicata region, which is mostly mountainous, there is a deep-seated tradition of dairy products from small ruminants reared in extensive and semiextensive systems, expressing the interaction among the environment, animals, and human practices (19). The Special Area of Conservation “Monti Foy” is interesting in terms of biodiversity maintenance. However, the misuse of pasture resources can affect the balance of the entire system (20). The mountainous area is characterized by a semiextensive livestock system, with local breeds being reared at pasture, resulting in overgrazing situations in summer, at a mean stocking rate of 6 LU ha^−1^ year^−1^, and undergrazing in other seasons. This grazing system, in addition to the expansion of plants indicators of pasture degradation such as thistles (*Cirsium arvense, Carduus* spp.), asphodels (*Asphodelus ramosus*), ferns (*Pteridium aquilinum*), and brambles (*Rubus fruticosus*), has led to the worsening of the grassland composition.

The diet of grazing animals, especially sheep and goats, varies according to the season due to the plant species available for grazing, the plants' phenological stage, climate conditions, and feeding behavior (plants and aerial parts browsed by animals) (21). This diversity affects the content of volatile compounds in milk and cheese, particularly the presence and abundance of molecules that affect flavor and aroma (22, 23). These volatile compounds are found in greater amounts in milk and dairy products when the animals are fed at pasture, particularly when they browse dicotyledons (15, 24–27). In addition, several studies have shown that ruminant products from grazing systems show variation in the content of beneficial compounds, such as particular classes of fatty acids (FAs), phenols, and vitamins A and E, and a higher degree of antioxidant protection (DAP), and that these contents are higher overall than in products from housed animals. In particular, the increase in FAs of healthy interest in milk occurs already 3 days after the abrupt transition from indoor to pasture diet (28). Furthermore, these products are perceived more positively by consumers because of their richer sensory profile (29, 30).

Vast areas of rangelands across the world are being grazed with increasing intensity. The interactions between livestock production and grassland biodiversity and conservation are debated (1); however, their connections with the quality of animal products have been less focused so far. The main aim of this work is to provide information for a discussion, based on published scientific studies, on (a) grassland biodiversity and conservation, (b) mountain dairy product quality, and (c) interactions between them in a specific mountain area. The discussion aims to lead toward a hypothesis for a revaluation of the traditional management system of the mountain agrosilvopastoral production chain, which is able to produce high-quality food and maintain and enhance grassland biodiversity.

## Case Study 1: Grazing System, Grassland Biodiversity, and Conservation

At the experimental farm (1,230 m a.s.l.) of the CREA–Research Center for Animal Production and Aquaculture in the municipality of Potenza (southern Italy), several studies have been carried out on the relationships between the grazing behaviors of local breeds and pasture biodiversity. The farm is included in the mountain Special Area of Conservation “Monti Foy” (40° 37′ N, 15° 42′ E) (defined by EU Habitats Directive 92/43/EEC), which is included in the list of Sites of Community Importance in the Mediterranean biogeographical region (IT9210215). In this area, the semiextensive livestock system is based mainly on local breeds (Garganica and Capra di Potenza goat breeds and Gentile di Puglia and Merino-derived sheep breeds). In the routine management of the experimental farm, sheep were fed at pasture with 2.2 LU ha^−1^ year^−1^ stocking rate, whereas goats were reared at 2.1 LU ha^−1^ year^−1^ stocking rate in separate fields.

A recent study (20) aimed to evaluate the effect of different stocking rates on the botanical parameters of natural pastures. Dry and pregnant Gentile di Puglia sheep were assigned to the permanent natural pasture previously grazed by goats for over 25 years, with an average potential yield of 5 t ha^−1^ year^−1^ (rich pasture). Ewes were allotted to three groups and assigned to three plots, characterized by Natura 2000 habitat 6210 seminatural dry grasslands *Festuco-Brometalia* (plot 1) and Natura 2000 habitat 6510 *Lowland hay meadows* (plots 2 and 3), with stocking rates of 0.2 LU ha^−1^ year^−1^ (plot 1), 4.0 LU ha^−1^ year^−1^ (plot 2), and 6.0 LU ha^−1^ year^−1^ (plot 3), the two limits indicated by the EU Standard 4.6 and an overgrazing situation (20). The animals grazed 8 h per day from early May to late September, sheltered overnight, and received pasture hay *ad libitum* as dietary supplementation to the grazing intake. The hay was produced from an area in the same farm, out of the three plots, characterized by seminatural dry grasslands *Festuco-Brometalia*. In the plots, visual assessment was carried out on seven functional groups: grasses, legumes, other species, palatable vs. unpalatable plants, thorny species, shrub species, and bare soil (expressed as percentage of coverage). The study on grazing behavior and the effect on grassland composition, combined with the results of the degradation of vegetation and biodiversity, revealed the limits of the monospecies flock mostly in the undergrazed plot (0.2 LU ha^−1^ year^−1^). Plot 1 showed a decrease in palatable species (from 98 to 85%) and a proportional increase in unpalatable and thorny species. Thorny species (*Carduus* sp.) increased from rare to >20%, with *Crataegus monogyna* (hawthorn) and *Ononis spinosa* increasing up to 20–25% in comparison to the level under the previous grazing management system (grazing goats with a stocking rate of 2.1 LU ha^−1^ year^−1^). Plot 2 showed the best effectiveness for the maintenance of the grassland botanical composition, with palatable species (30% each for grasses, legumes, and others) unvarying at 90%, thorny (thistles) species at <5% and unpalatable (ferns) species at 5%. In plot 3, a severe drop of the palatable species was observed in summer, as well as increase in bare soil (from 0 to 30%) and increase in/appearance of thistles/asphodels. Afterward, the area was interested by a great fire (summer 2017) during the 6th year of grazing by solely sheep; the extension of the event was explained also with the missing pruning of the bushes, usually done by grazing goats, and the abundance of dry grass in the undergrazed areas (unpublished data).

## Case Study 2: Quality of Dairy Products From Grazing System

Studies were conducted at the CREA experimental farm to evaluate the effect of feeding at pasture compared with other feeding treatments on volatile organic compounds (VOCs), FAs, α-tocopherol, retinol, and DPA in goat milk and cheese. VOC content was assessed by multiple dynamic headspace extraction and gas chromatography (GC)–mass spectrometry (31). FA separation and quantification were carried out using a GC, as reported by Di Trana et al. (32), and fat-soluble vitamins and DPA were assessed according to Pizzoferrato et al. (33). Local Mediterranean Red breed goats were used. A first study evaluated the VOC content and profile in the milk of goats fed (a) at pasture (grazing), (b) pasture hay harvested from the same grazing area, and (c) mixed hay (alfalfa, perennial rye grass, and orchard grass). The difference observed among the three diets could be linked to the contributions of the different plant species measured in the diet (Table 1). Milk from the grazing goats showed significantly higher monoterpene and sesquiterpene content than milk from the goats fed on pasture hay and mixed hay. The contribution of forbs (38%) might explain the result.

**Table 1:** Monoterpenes and sesquiterpenes content (mean ± SEM) in milk from three feeding systems [from (34)].

**Feeding treatment**	**Grazing**	**Pasture hay**	**Mixed hay**	
**Plants category in the diet (%)**				
Grasses	40	36	40	
Legumes	22	33	50	
Forbs	38	31	10	
**Milk VOC (ng/L)**				
Monoterpenes	2,031.0^a^ ± 429	1,374.0^a^ ± 226	718.0^b^ ± 154	*P* < 0.05
Sesquiterpenes	4,480.0^a^ ± 626	2,334.0^b^ ± 324	610.0^c^ ± 152	*P* < 0.05

a, b, c *Letters mean significant difference among means. The significance of the diet botanical composition (plants category) was not detected*.

**Figure 1:**
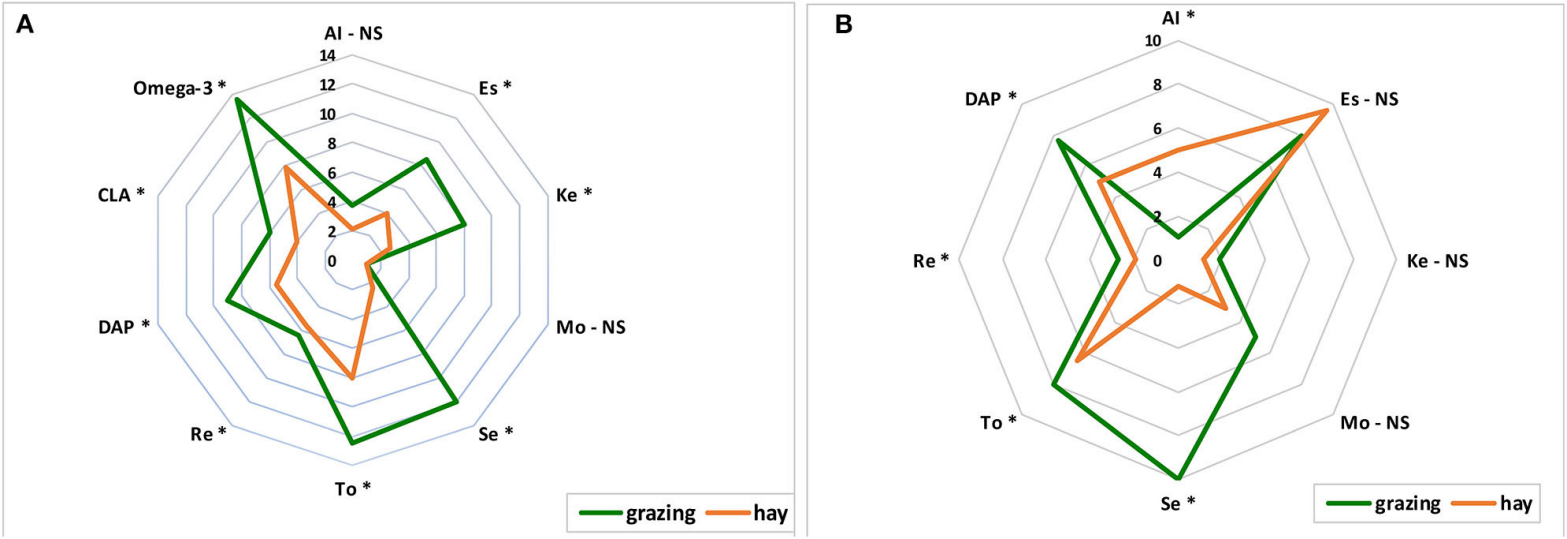
Qualitative profile of goat milk **(A)** and cheese **(B)** from grazing and hay feeding system [from (35)]. Al, alcohols; Es, esters; Ke, ketones; Mo, monoterpenes; Se, sesquiterpenes; To, tocopherol; Re, retinol; DAP, degree of antioxidant protection; CLA, conjugated linoleic acid. *P < 0.05; NS = not significant.

A second study was carried out in the same area to examine the effect of pasture vs. indoor feeding systems during winter, spring, and summer on α-tocopherol and retinol, FA content, and DPA in goat milk and cheese. Two homogeneous groups were used: goats grazing 8 h per day on native herbaceous pasture (G) and goats housed and fed *ad libitum* with hay harvested from the same native pasture (H), both supplemented with concentrate feed (600 g/head per day at 13% CP). The results showed that the qualitative profiles of milk and cheese were very different between the G and H groups throughout the seasons (Figures 1A,B). Tocopherol and retinol increased in milk by 61.3 and 20.0% in the G and H groups, respectively. The same trend was observed for DAP; this index was 61.6% higher in milk from grazing goats than in milk from the housed goats fed hay. Highly significant differences between the milk fat quality of the G and H groups were detected. In fact, conjugated linoleic acid (CLA) and ω-3 FA content were higher in milk from goats grazing on native pasture than in milk from housed goats (Figure 1A). The cheese quality almost completely reflected the milk quality. Cheese produced from the G group goat milk was richer in sesquiterpenes, tocopherol, and retinol than cheese produced from the H group milk; similarly, the DAP index was higher in cheese from the G group than in cheese from the H group (Figure 1B). The results confirmed that feeding on a grazing basis conferred higher total quality on milk and cheese than the housing feeding system throughout the whole grazing season.

## Discussion

### Feeding Management and Grassland Biodiversity and Conservation

The results of the stocking rate case study 1. Sepe et al. (20) are in agreement with Petz et al. (36), who identified three livestock stocking rate categories at pasture, indicated by the authors as “grazing intensities”: low (0.0–0.4 LU ha^−1^ year^−1^), moderate (0.4–0.6 LU ha^−1^ year^−1^), and high (0.6–1.0 LU ha^−1^ year^−1^) grazing intensities, calculated as the ratio between biomass grazed and biomass available for grazing. The results showed that, on average, only 4.2% of the biomass produced annually was consumed by livestock. Erosion prevention was 10% lower in areas with high grazing intensity than in areas with low grazing intensity. Therefore, the authors found lower biodiversity values, lower capacity for erosion prevention, and unsustainable forage utilization in high-grazing-intensity areas. The case study 1 results reported in this article agreed with Petz et al. (36) that grazing systems, when adequately managed, can contribute to the maintenance of botanical biodiversity. The results reported here supported by the aforementioned studies on goat grazing behavior (17, 21), together with the elements of the traditional management system in that area, led the authors to grazing practice recommendations that include the use of local-breed sheep and goats because they are capable of exploiting natural resources in a sustainable manner that protects the environment [as emerged from previous studies reviewed by (18)]. The authors advised a stocking rate of 4.0 LU ha^−1^ year^−1^ to avoid limit situations (undergrazing or overgrazing) in the case of rich pasture and to contribute to the maintenance of grassland biodiversity and conservation, the main reason for which dairy products from mountain systems show high-quality standard, as discussed in the following subsection.

### Quality of Milk and Cheese From Pasture-Based Diets

Overall, goat products from grazed herbage revealed higher-quality values, for example, in monounsaturated FA and polyunsaturated FA (PUFA), which are beneficial for human nutrition, and higher total consumer acceptability of cheeses (37). A study on goats grazing on native pasture compared to stall-fed goats revealed an increase in the CLA and ω-3 contents achieved in the milk of goats fed at pasture (32). Moreover, the docosahexaenoic acid and eicosapentaenoic acid content reached interesting levels in the milk fat of grazing goats that may be linked to the content of precursors in the diet, such as long-chain omega-3 PUFA. These results agree with Decandia et al. (38), who found higher CLA and VOC content, particularly ketones and aldehydes, in the milk of goats browsing a Mediterranean lentisk-based shrubland than in the milk of housed goats. Diminishing amounts of fresh grass percentages in the diet of Camosciata goats led to significant decreases of vaccenic, rumenic, and α-linolenic acids in milk, thus determining a worsening of the health value of milk fat associated with an increase in the percentages of hypercholesterolemic saturated FAs (39). A sudden transition of dairy Valdostana goats from winter indoor to pasture-based diets significantly affected the concentrations of FA in milk already 3 days after the diet change. In milk short- and medium-chain FA rapidly decreased after transition, whereas the sum of CLA isomers and omega-3 FAs markedly increased (28). A study conducted in Northern Europe confirmed that the milk from grazing goats had significantly higher fat, protein, and total nonfat solids than the milk from goats kept indoors (40). Grazing caused significantly higher concentrations of vitamin A and D_3_ than in the milk from goats fed hay. For goats on grass diet, the rumenic acid and *n*−3 FA contents of the milk increased significantly. Additionally, the *n*−6/*n*−3 ratio in the milk from goats fed grass was significantly lower than that in the milk from goats fed indoor.

Several investigations have reported that the diet ingested by goats influenced milk and cheese polyphenol content. An increase in the total polyphenol content in goat milk and cheese was obtained from grazing animals compared with stall-fed goats (41). These results are in agreement with Cabiddu et al. (42) and Chávez-Servín et al. (43), who observed a feeding system effect (free-range grazing and indoor-fed animals) on phenolic compounds and antioxidant capacity in goat milk, whey, and cheese.

A large study has highlighted the predominant effect of pasture-based diets compared to rations based on hay on the content of fat-soluble carotenoids and vitamins in milk and cheese (44). Pasture-based rations were associated with higher levels of xanthophyll, retinol, α-tocopherol, and total antioxidant capacity (TAC) in cheese than hay-based rations, whereas in milk and cheese a higher percentage of concentrates in the herd diet led to lower xanthophyll and α-tocopherol contents (15, 37, 44, 45).

Regarding VOC content and profile, goats fed with fresh and different meadow species transmit different characteristics to Caciotta cheese that are also perceivable on a sensorial level (22). Seasonal variations in the availability and quality of grazing grass influence the quantitative and qualitative content of VOC compounds in cheese obtained from grazing goats (31, 42, 46). Some volatile compounds, e.g., terpenes, can be used as biomarkers because they can be transferred from herbage to milk and contribute a characteristic flavor to the cheese. Terpenoids and FAs were found to be valuable as chemical fingerprint for the characterization of the dairy cows' feeding regimen (47). Indeed, the authors suggested that coupling terpenoids and FAs information could be suitable for tracing Asiago d'Allevo PDO cheeses produced during the early and late summer grazing and the autumn/winter indoor seasons.

The odor profiles of milk and cheeses were explained in a study where milk and cheese showed significant differences over three seasons, especially in ketones, alcohols, and ester compounds (46). The detection of sesquiterpenes could be extremely useful in distinguishing whether a cheese has been produced with milk from animals fed on pasture or with the total mixed ration system (48). In this context, the traceability of products obtained from grazing animals compared to stall-fed animals represents an ongoing current objective. Future directions converge toward the development of a tool or procedure based on scientific parameters that in synthesis shows indications of the origin of the product and its healthy quality.

Pizzoferrato et al. (33) developed the DAP index, calculated as the molar ratio between an antioxidant compound and a selected oxidation target. It evaluated goat cheese resistance to oxidative reactions. It is noteworthy that DAP values in goat products were 10-fold higher in grazing goats than in stall-fed goats. The DAP index was able to distinguish dairy products when the grazed herbage in the goats' diet exceeded 15%. These results agree with Delgadillo-Puga et al. (49) and Cabiddu et al. (42), who found an increase in PUFA, DAP, and phenol content in the milk of goats reared in shrubland compared to stall-fed goats.

Recently, a new index, the General Health Index of Cheese (GHIC), was developed by Giorgio et al. (50); this index combines in a single value the contributions of several components to cheese quality. It takes into account different indicators of products obtained from animals fed with fresh forage or at pasture: polyphenols, CLA isomers, PUFA, omega-3 FA, and TAC. In addition to CLA, PUFA, and omega-3, which are already known to be health-promoting compounds, polyphenols and total antioxidant capacity were used in GHIC calculations because of health researchers' increasing interest in these compounds. The GHIC index, which combines the positive components found in cheese, seems to distinguish cheeses obtained from different fresh forages.

Dairy products from the grazing system, compared to those from the indoor-fed supplementation strategy, carry a real added value because of their impact on human health because of their higher content of beneficial metabolites (30), as well as the hedonistic and sociological aspects.

The authors refer to the role of small ruminant grazing in the framework of the Millennium Assessment (51). There, the relationship between feeding at pasture and biodiversity is included in the provisioning of habitat services because grazing facilitates the life cycles of animals and plants, prevents the occurrence of less valuable ecological states through the encroachment of bush and/or invasive species, and conserves wildlife and protected areas in coevolved landscapes. In the most important cluster of habitat services, grazing systems support the maintenance of species life cycles and the connection of habitats. The Millennium Assessment showed that “with appropriate actions, it is possible to reverse the degradation of many ecosystem services over the next 50 years, but the changes in policy and practice required are substantial and not currently underway.”

## Conclusions

The livestock system based on grazing local breeds can provide benefit to both the environment and the mountain population, given the habitat service that it provides. Two case studies were presented in this article with the aim of presenting two issues concerning the mountain system that are usually considered separately. Combining the outcomes of the aforementioned studies, the authors recommend a management system that revalues the traditional approach. This system, which has traditionally proven to be more sustainable and respectful of the mountain environment, consists of (i) mixed flocks of local breeds of small ruminants, sheep, and goats, in variable percentages (up to 80% sheep and 20% goats); (ii) grazing system with stocking rates ranging from 2.1 to 4.0 LU ha^−1^ year^−1^; (iii) supplementation of diet, during lactation, with native pasture hay and concentrated feed. This management system, in comparison with sheep-only herds, allows high-quality dairy products even in summer, when sheep are in a dry stage (physiological stage after lactation). The transferability of this system to other, similar Mediterranean areas would be limited only by the yield of the pasture. On less rich pastures, the recommended stocking rate would be reasonably lower, i.e., 0.2 LU ha^−1^ year^−1^.

In the mountain livestock system of Monti Foy, the management system recommended in the present article would contribute over time to grassland biodiversity preservation, in addition to preventing fire. In addition, milk and cheese from the grazing system are richer than those from the housed animals feeding system, mainly owing to the higher content of healthy compounds, as well as the hedonistic characteristics. When the relationship between grassland biodiversity maintenance and this quality is taken into account, these products appear worthy of being valued and sold at higher prices, which is a viable way to reward farmers who sustain the struggle to live and produce in mountain areas and encourage them to continue their work and not give up in these tough but incomparable production systems. Finally, the mountain management system recommended in the present article, inspired by the traditional system, offers an approach for mountain area land managers, a viable way to produce high-quality food together with preserving the system.

As a new perspective, further research could aim to find new markers/indicators of the high quality of the products from local breeds in grazing system and more strictly relate them to the mountain system. This request often comes from the stakeholders (farmers/cheesemakers). To this end, a multidisciplinary study may be a viable approach, involving countries in the Mediterranean area with similar mountain systems, to address the complex relation among grassland biodiversity, livestock breeding, and livestock products. The evaluation of those markers would concur with the development of an economic model that can recognize and assign the added value, thus supporting and protecting production systems that would otherwise be less competitive and less economically sustainable.

## Data Availability Statement

The original contributions presented in the study are included in the article/supplementary material, further inquiries can be directed to the corresponding author/s.

## Author Contributions

SC for conceptualization, resources, review, final review, and supervision. MM for analysis of resources, writing—original draft. AD for writing and final review. LS for conceptualization, resources, writing, review, final review, and editing. All authors contributed to the article and approved the submitted version.

## Conflict of Interest

The authors declare that the research was conducted in the absence of any commercial or financial relationships that could be construed as a potential conflict of interest.
